# To culture or not to culture: careful assessment of metabarcoding data is necessary when evaluating the microbiota of a modified-atmosphere-packaged vegetarian meat alternative throughout its shelf-life period

**DOI:** 10.1186/s12866-022-02446-9

**Published:** 2022-01-25

**Authors:** E. Duthoo, K. De Reu, F. Leroy, S. Weckx, M. Heyndrickx, G. Rasschaert

**Affiliations:** 1 Flanders Research Institute for Agriculture, Fisheries and Food (ILVO)– Technology and Food Science Unit, 9090 Melle, Belgium; 2 Research Group of Industrial Microbiology and Food Biotechnology (IMDO), Faculty of Sciences and Bioengineering Sciences, Vrije Universiteit Brussel, Brussels, Belgium; 3 Department of Pathology, Bacteriology and Avian Diseases, Ghent University, B-9820 Merelbeke, Belgium

**Keywords:** Vegetarian charcuterie, Microbial ecology, Spoilage

## Abstract

**Background:**

As the increased consumption of ready-to-eat meat alternatives is a fairly recent trend, little is known about the composition and dynamics of the microbiota present on such products. Such information is nonetheless valuable in view of spoilage and food safety prevention. Even though refrigeration and modified-atmosphere-packaging (MAP) can extend the shelf-life period, microbial spoilage can still occur in these products. In the present study, the microbiota of a vegetarian alternative to poultry-based charcuterie was investigated during storage, contrasting the use of a culture-dependent method to a culture-independent metagenetic method.

**Results:**

The former revealed that lactic acid bacteria (LAB) were the most abundant microbial group, specifically at the end of the shelf-life period, whereby *Latilactobacillus sakei* was the most abundant species. Metabarcoding analysis, in contrast, revealed that DNA of *Xanthomonas* was most prominently present, which likely was an artifact due to the presence of xanthan gum as an ingredient, followed by *Streptococcus* and *Weissella*.

**Conclusions:**

Taken together, these results indicated that *Lb. sakei* was likely the most prominent specific spoilage organisms (SSO) and, additionally, that the use of metagenetic analysis needs to be interpreted with care in this specific type of product. In order to improve the performance of metagenetics in food samples with a high DNA matrix but a low bacterial DNA load, selective depletion techniques for matrix DNA could be explored.

**Supplementary Information:**

The online version contains supplementary material available at 10.1186/s12866-022-02446-9.

## Introduction

Although meat is still a staple food in the diet of the average Belgian consumer, a market study commissioned by the Flanders’ Agricultural Marketing Board (VLAM) has noted that, in 2019, 23% of the Belgian population consumed meat substitutes at least once a week [1]. Also, approximately 13 million metric tons of alternative proteins were consumed globally in 2020 [2]. Meat substitutes have become more available as the food industry increasingly develops and produces a variety of such products [3]. As the broadening range of commercially available products is fairly recent, such as the Beyond Burger, the Impossible Burger and Quorn among others, not much research has been conducted on their associated microbiota, especially when compared to the available data obtained for meat and meat products. Yet, this information is indispensable, considering the potential influence of microorganisms on both food safety, *e.g. Listeria monocytogenes*, and the shelf-life period, *e.g. Pseudomonas* or LAB [4], even if chilled modified-atmosphere packaging (MAP) allows for an extended shelf-life period [5]. Generally, most of the available information on meat (product) alternatives is based on the microbial investigation of soy-based foods, such as tofu and tempeh [6–8]. These products are restricted to a small niche market in the West and have characteristics that differ from the product analyzed in this study, i.e., they are fermented and completely plant-based (whereas the vegetarian meat alternative in this study also contains ingredients derived from eggs), making it difficult to draw similarities between the product at hand.

Evaluation of food spoilage is usually monitored by culture-dependent methods. These methods involve cultivation on selective agar media, followed by molecular techniques to further identify bacteria to genus or species level [4, 9]. The sequencing of the bacterial 16 S rRNA gene is a valuable tool in identifying species, as this gene has both conserved and variable regions, making it possible to use primers that target all bacteria and span species-specific sequences [4]. However, these methods have a limited potential for the characterization of specific spoilage organisms (SSO) in food products due to their low-throughput nature and limits of detection [10, 11]. They may underestimate SSO diversity in terms of both species richness and abundance, as culturable microorganisms may only represent a small fraction of the total microbiota [10]. The recent developments in high-throughput sequencing such as metabarcoding, otherwise known as metagenetics, can offer additional insights [12]. This methodology also focuses on the 16 S rRNA gene, in the case of Archaea and Bacteria, and involves DNA isolation directly from the matrix, which then undergoes targeted amplification of one or a few variable regions of the gene followed by sequencing the obtained amplicons. This way, a cost-effective overview of the taxonomic composition of a sample can be obtained at a higher through-put, allowing to assess the presence of the microorganisms involved in food spoilage [11].

In this study, the composition and dynamics of the microbiota of a vegetarian alternative to poultry-based charcuterie was investigated during storage using both culture-dependent and culture-independent methods. Samples were collected just before the products were sliced, shortly after slicing and at the end of the shelf-life period and identified microbiota were compared. The aim of the study was to uncover potential SSO’s present in this product during its shelf-life period and to obtain novel information on the diversity and progression of the most abundant microorganisms during shelf life using the metagenetics method.

## Materials and Methods

### Sample collection and storage

**Fig. 1:**
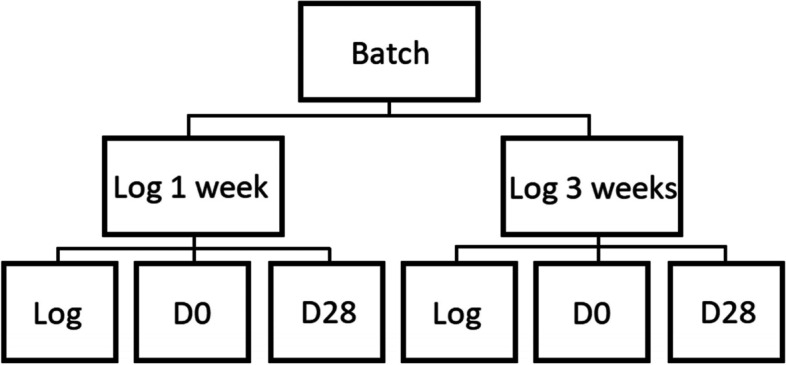
Schematic overview of sample collection per batch. Five batches were analyzed, at six samples per batch, resulting in a total of 30 samples. Log describes the sampling of the unsliced meat log. Log 1 week and Log 3 weeks represent when a vegetarian product log was sliced (i.e. vegetarian product logs sliced one and three weeks after production, respectively). D0 describes that the sliced product was analyzed on the day of slicing, while D28 represent the sliced product which was analyzed after 28 d of storage

In this study, curry-flavored vegetarian slices, composed out of egg white (38%), water (37%), sunflower oil (15%), salt (2.7%) and 1.6% curry spices, and also containing <2% for each of the following individual ingredients: bamboo fibers, spices, sugar, thickeners, acidity regulators and colorings, were sampled. The product was pressed into a vegetarian product log which was subsequently cooked, reaching a core temperature of ≥72 °C. Sample collection was performed at the moment of slicing and packaging, under MAP conditions (70% N_2_, 30% CO_2_), within the premises of a food business operator (FBO), as previously described [13]. Each batch of the product was sampled twice to reflect the potential microbial variability preceding slicing; i.e. when a vegetarian product log was sliced one week after production and when a different vegetarian product log of the same batch was sliced at the end of the unsliced vegetarian product log’s shelf-life period two weeks later, so 3 weeks after production (Fig. 1). Day-0 samples are samples taken from the sliced and packaged product, analyzed on the day of slicing. The 28-day storage entailed 19 days at 7 °C, followed by 9 days at 8 °C, as is performed in shelf life studies by the FBO. A total of five batches were analyzed, resulting in 30 samples. Samples were transported to the laboratory (with a transportation period of maximum 30 min) under cooled conditions using a cooler with cooling blocks.

The results of the culture-dependent methods indicated potential cross-contamination from different products sliced on the same slicing line. Based on these results, it was decided to conduct an environmental sampling at the premises of the FBO. The limitation of the approach was, however, that this was performed eight months after the sampling of the products, requiring careful interpretation of the data as some shift in microbiota cannot be excluded. During production, 4 environmental samples of the food contact surfaces of the slicer used for the curry-flavored vegetarian meat alternative were taken. Sponge-sticks (3 M™, St.Paul, MN, USA), moistened with 10 ml of maximum recovery diluent (MRD, Oxoid, Basingstoke, Hampshire, United Kingdom), were used to perform the surface sampling (approximately 625 cm² each).

### Microbiological culture-dependent analysis

Ten grams of each sample were collected and analyzed, as previously described [13]. Plate count agar (PCA; Oxoid, Basingstoke, Hampshire, United Kingdom) was used for the enumeration of presumptive total psychrotolerant and psychrophilic aerobic (TAC); incubated at 21 °C for 5 d. Reinforced clostridial agar (RCA; Oxoid, Basingstoke, Hampshire, United Kingdom) was used for the enumeration of presumptive total psychrotolerant and psychrophilic anaerobic count (TANC) and incubated at 21 °C for 5 d. For the enumeration of presumptive psychrotolerant and psychrophilic lactic acid bacteria (LAB), de Man, Rogosa and Sharpe agar (MRS; Oxoid, Basingstoke, Hampshire, United Kingdom) of pH 6.5 and M17 agar (Oxoid, Basingstoke, Hampshire, United Kingdom) were both incubated at 21 °C for 5 d. For the enumeration of presumptive yeasts and moulds, oxytetracycline-glucose-yeast extract agar (OGYE; Oxoid, Basingstoke, Hampshire, United Kingdom) was incubated at 25 °C for 5 d. For specific enumeration of *Enterococcus* spp., Slanetz and Bartley agar (S&B; Oxoid, Basingstoke, Hampshire, United Kingdom) was incubated at 37 °C for 48 h; and colonies were confirmed by testing for growth at 44 °C, in 40% bile and performing an additional catalase test. Streptomycin thallous acetate actidione agar (STAA; Oxoid, Basingstoke, Hampshire, United Kingdom) was incubated at 21 °C for 48 h for the enumeration of presumptive *B. thermosphacta*; and confirmation steps were carried out. For the enumeration of presumptive sulfite-reducing clostridia, tryptose sulphite cycloserine agar (TSC; Oxoid, Basingstoke, Hampshire, United Kingdom) was incubated at 37 °C for 24 h. For the enumeration of presumptive *B. cereus*, Mannitol egg yolk polymyxin agar (MYP; Oxoid, Basingstoke, Hampshire, United Kingdom) was incubated at 30 °C for 48 h; and confirmation steps were carried out. For the enumeration of presumptive Enterobacterales, Violet red bile glucose agar (VRBG; Bio Rad, Marnes-la-Coquette, France) was incubated at 37 °C for 24 h. One log colony forming units (CFU)/g was the lower limit for enumeration for all media.

Surface swab samples from the environment were analyzed for TAC, TANC and LAB counts on MRS, following the same method as described above.

The pH was measured using a pHenomal pH 2100 L in combination with a SF113 electrode (VWR, Leuven, Belgium). Water activity (a_w_) was measured for each food sample using the AQUALAB 4TE water activity meter (Metergroup, München, Germany).

### Isolate collection

Isolates were collected at random from the PCA, RCA, and MRS agar media demonstrating the highest decimal dilution with bacterial growth, as previously described [13]. A median level of 33% of colonies were randomly picked from each of the three types of media. From PCA, RCA, and MRS agar, a total of 97, 83 and 64 isolates, respectively, were collected.

### Identification of isolates

Microbial DNA was extracted as previously described [14] from each isolate, except for 39 out of 244 isolates that could not be cultivated after storage at -80 °C. In short, this included suspension of pure cultures in lysostaphin and incubation at 37 °C. Then, proteinase K was added and incubation was performed at 60 °C for 10 min and subsequently at 100 °C for 5 min. (GTG)_5_ PCR fingerprinting was carried out on the obtained lysates: this technique is based on a PCR amplification of repetitive bacterial DNA elements, in this case using (GTG)_5_ elements [15, 16]. Using Bionumerics version 7.6 (Applied Maths, Sint-Martens-Latem, Belgium), the obtained fingerprints were clustered based on their similarity (based on UPGMA [unweighted pair group method with arithmetic averages algorithm]; optimization 1%; position tolerance 1%).

Based on the (GTG)_5_ fingerprint clusters, 43, 34 and 21 isolates (out of a total 82, 72, and 51 isolates originating from PCA, RCA and MRS agar, respectively) were selected as representative isolates of visually defined clusters grouping isolates with the same band patterns and were further identified. One representative isolate was selected for clusters containing up to three isolates, while a minimum of two isolates were selected for identification of clusters containing >4 isolates. As previously described [14], identification was based on the amplification of a 1127 bp-region of the 16 S rRNA gene of the selected isolates, using universal bacterial primers 16F27–1 (pA,5′-3′ sequence: AGA GTT TGA TCC TGG CTC AG) and 16R1522 (pH, 5′-3′ sequence: AAG GAG GTG ATC CAG CCG CA) [17]. Sanger sequencing was performed on the PCR products by Genewiz (Takeley, United Kingdom). The sequence reads were used to identify the isolates to their presumed species level by comparing them to the EZBioCloud Database, where species in the database were selected based on the highest identity (of at least 98.5%) and coverage. To calculate the biodiversity indexes, the Gleason-Margalef’s index was used [18].

From the food contact surfaces of the slicers, 56 isolates were identified (29 from PCA, 19 from RCA and 8 from MRS agar).

### Metabarcoding

Sample preparation preceding metabarcoding analysis was performed as previously described [13]. DNA extraction was performed using a DNeasy mericon Food Kit (Qiagen, Antwerp, Belgium), following the manufacturer’s instructions. The V3-V4 region of the 16 S rRNA gene was targeted to obtain the fragment libraries, which were amplified as described by the Illumina protocol and with primers of Klindworth et al. [19] on an Illumina MiSeq sequencer with 2 × 300 bp chemistry by a provider known as Admera Health (South Plainfield, NJ, USA). The following sample codes were used: V symbolizes vegetarian product, B indicates the batch number while L represents when a vegetarian product log was sliced (i.e. L1 and L3 represent vegetarian product logs sliced one and three weeks after production, respectively). D0 describes that the product was analyzed on the day of slicing, while D28 represent the product which was analyzed after 28 d of storage.

### Sequence read processing

Demultiplexing of the amplicon sequencing dataset was performed by the sequence provider and barcodes were clipped off. The processing pipeline was entirely performed in RStudio 1.3.1093. To remove primer sequences, the ShortRead package was used [20]. Then, the “filterAndTrim” function from the DADA2 package was used to quality filter, with a maximum expected error of 2 for the forward and 4 for the reverse reads, and trim the reads, where forward and reverse reads were trimmed to 280 bp and 210 bp, respectively [21]. After this, the forward and reverse reads were merged and the amplicon sequence variants (ASVs) count tables were calculated. The Ribosomal Database Project (RDP) naive Bayesian classifier [22] with SILVA v138 as reference database was used to assign taxonomy. The used version of SILVA does not take into account the recent reclassification of *Lactobacillus* encompassing genera *Companilactobacillus*, *Dellaglioa*, *Lacticaseibacillus*, *Lactiplantibacillus*, *Latilactobacillus*, and *Paucilactobacillus* [23]. The “rarecurve” function of the Vegan package was used to make rarefaction curves. The Phyloseq package was used to calculate Shannon-Wiener diversity and Chao1 richness indices. Based on the rarefaction curves, after removing mitochondria and chloroplasts, eleven out of thirty samples were not used in the downstream analyses because not enough reads were available to obtain a reliable view on the taxa present. The Bray Curtis dissimilarities were calculated between all samples and non-metric multidimensional scaling plots (NMDS ordination plots) were constructed using phyloseq to compare the bacterial diversity between conditions [24].

### Statistical analysis

RStudio Version 1.3.1093 (R Core Team, R Foundation for Statistical Computing, Vienna, Austria) was used to perform analysis of variance (ANOVA, confidence interval 95%) and Tukey’s test, to verify potential significant differences between enumerations (p-value < 0.05).

## Results

### Microbiological analysis

**Fig. 2:**
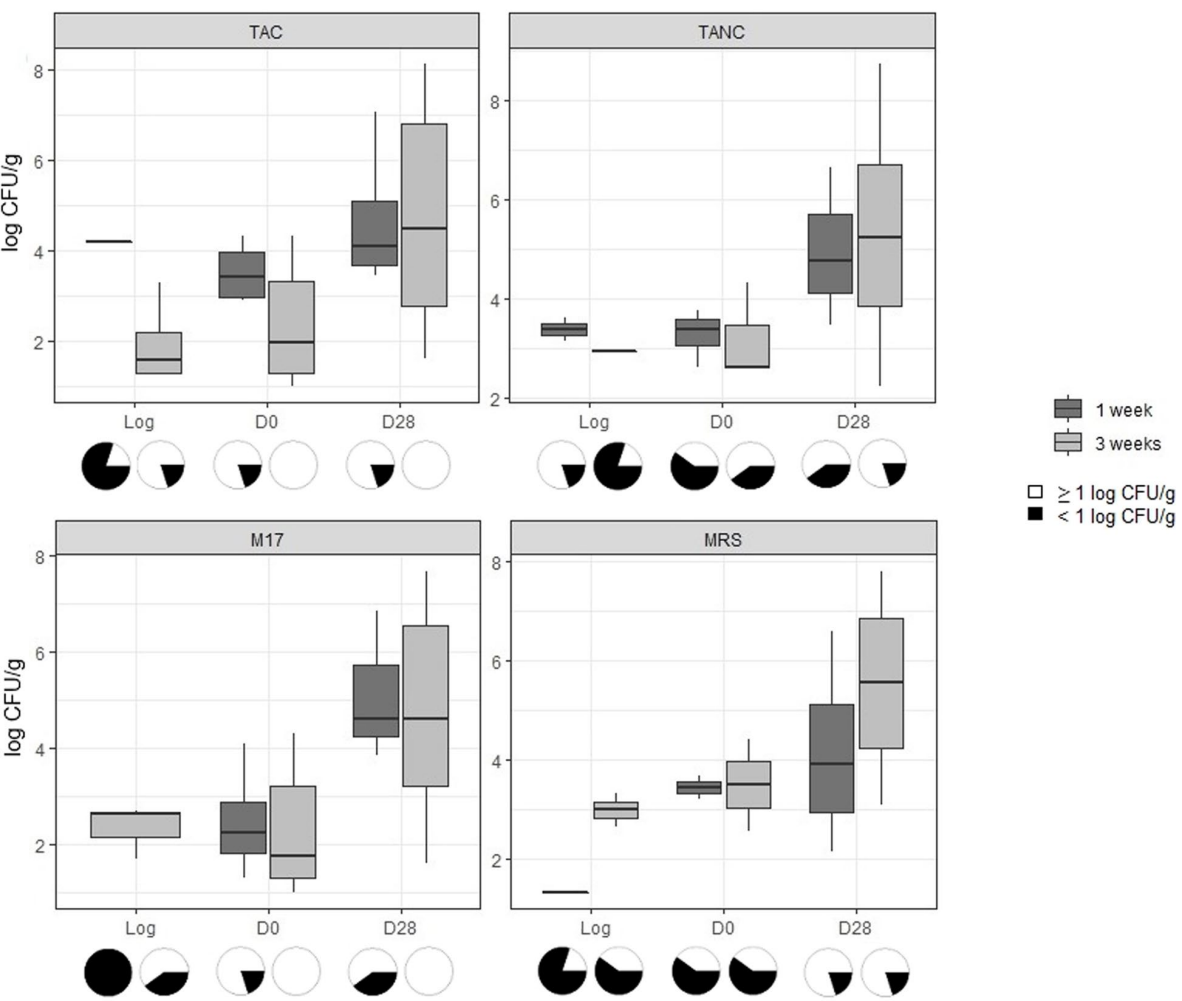
Enumerations for total aerobic, anaerobic and lactic acid bacteria counts. Log CFU/g values for total psychrotolerant and psychrophilic aerobic (TAC) and anaerobic counts (TANC) and lactic acid bacteria counts (M17 and MRS agar) found on curry-flavored vegetarian product samples. Each boxplot contains five data points, except in case a sample was below the enumeration limit of 1 log CFU/g, in which case, n will deviate. The boxplots represent the 1st quartile, median and the 3rd quartile of each dataset. Pie charts under the graphs visualize the deviation in data points: countable samples are represented by white and samples below the enumeration limit are represented by black. Boxplots within a graph, indicated by the same letter signify no significant differences between the results

Bacterial loads on the curry-flavored vegetarian meat alternative were estimated for TAC, TANC and LAB using samples obtained from the 1-week old vegetarian product log, the 3-weeks old vegetarian product log and after slicing and storage of these respective products (Fig. 2; Additional Table 1). Tukey’s test indicated no significant difference in bacterial numbers between the sliced day 0 and day 28 products of 1-week old vegetarian product logs and 3-weeks old vegetarian product logs, respectively.

**Table 1:** Average (in bold) and standard deviation of pH and a_w_ values at three different production stages: the unsliced vegetarian product log, at the day of slicing (D0) and at the end of its shelf life (D28)

	pH	a_w_
*1 week*		
Unsliced log	**5.47** ± 0.10	**0.9716** ± 0.0012
D0	**5.41** ± 0.15	**0.9727** ± 0.0035
D28	**5.43** ± 0.13	**0.9706** ± 0.0032
*3 weeks*		
Unsliced log	**5.51** ± 0.10	**0.9772** ± 0.0113
D0	**5.44** ± 0.15	**0.9737** ± 0.0032
D28	**5.29** ± 0.48	**0.9723** ± 0.0046

The counts for TAC, TANC and LAB at the end of the shelf-life period deviated between 4.1 and 5.5 log CFU/g and were not significantly different from each other at each examined sampling moment. Yeast and fungi were only found once on a vegetarian product log sample, at 1.0 log CFU/g (Additional Table 2). Other microbiological parameters, including sulphite-reducing clostridia, Enterobacterales, *Bacillus cereus*, *Brochothrix thermosphacta* and *Enterococcus* spp., were rarely countable (Table 2).

**Table 2:** Genus and presumptive species identity of isolates for the curry-flavored vegetarian slices samples from PCA, RCA and MRS agar media. Confidence intervals for percentages of number of identified isolates for the most abundant identifications per sample type

Presumptive species	Isolates from vegetarian slices (*n*=205)								
	**Unsliced log (** ***n*** **=19)**			**D0 (** ***n*** **=72)**			**D28 (n=114)**		
	**PCA** ***n*** **=10**	**RCA** ***n*** **=5**	**MRS** ***n*** **=4**	**PCA** ***n*** **=38**	**RCA** ***n*** **=23**	**MRS** ***n*** **=11**	**PCA** ***n*** **=34**	**RCA** ***n*** **=44**	**MRS** ***n*** **=36**
**Gram positive (n=178)**									
*Bacillus licheniformis*	10%	0	0	0	0	0	0	0	0
*Bacillus subtilis*	10%	0	0	0	0	0	0	0	0
*Bacillus tequilensis*	0	0	0	3%	0	0	0	0	0
*Bacillus zhangzhouensis*	10%	0	0	0	0	0	3%	0	0
*Buttiauxella agrestis*	0	0	0	0	4%	0	0	0	0
*Carnobacterium divergens*	0	0	0	0	0	0	21% [7-34]	9% [0-18]	19% [7-32]
*Carnobacterium inhibens*	0	0	0	3%	0	9%	0	0	0
*Carnobacterium maltaromaticum*	10% [0-29]	0	0	5% [0-12]	30% [12-49]	18% [0-41]	3% [0-9]	11% [2-21]	0
*Enterococcus avium*	0	0	0	0	0	0	0	0	8% [0-17]
*Enterococcus devriesei*	0	0	0	0	4%	0	0	0	0
*Enterococcus gilvus*	0	0	0	0	0	0	3%	0	3%
*Enterococcus malodoratus*	0	0	0	0	0	9% [0-26]	0	16% [5-27]	3% [0-8]
*Enterococcus viikkiensis*	0	0	0	0	0	0	3%	0	0
*Erysipelothrix* spp.	0	0	0	3%	0	0	0	0	0
*Glutamicibacter bergerei*	0	0	0	3%	0	0	0	0	0
*Granulicatella adiacens*	0	0	0	0	4%	0	0	0	0
*Latilactobacillus sakei*	10% [0-29]	100%	100%	21% [8-34]	30% [12-49]	64% [35-92]	47% [30-64]	59% [45-74]	67% [51-82]
*Leucobacter luti*	0	0	0	3%	0	0	0	0	0
*Leuconostoc carnosum*	10% [0-29]	0	0	0	0	0	0	0	0
*Micrococcus luteus*	0	0	0	0	0	0	3%	0	0
*Paenibacillus polymyxa*	0	0	0	0	0	0	3%	0	0
*Paenibacillus taohuashanense*	10%	0	0	0	0	0	0	0	0
*Staphylococcus hominis*	10%	0	0	0	0	0	0	0	0
*Staphylococcus saprophyticus*	10%	0	0	11%	0	0	0	0	0
*Vagococcus fluvialis*	10%	0	0	0	0	0	0	0	0
*Vagococcus silagei*	0	0	0	0	0	0	9% [0-18]	5% [0-11]	0
**Gram negative (n=27)**									
*Acinetobacter albensis*	0	0	0	5%	0	0	0	0	0
*Comamonas jiangduensis*	0	0	0	3%	0	0	0	0	0
*Haemophilus piscium*	0	0	0	3%	4%	0	0	0	0
*Morganella psychrotolerans*	0	0	0	0	4%	0	0	0	0
*Providencia burhodogranariea*	0	0	0	3%	0	0	0	0	0
*Providencia rustigianii*	0	0	0	0	4%	0	0	0	0
*Pseudomonas bubulae*	0	0	0	3%	0	0	0	0	0
*Pseudomonas caeni*	0	0	0	3%	0	0	0	0	0
*Pseudomonas versuta*	0	0	0	3%	0	0	3%	0	0
*Psychrobacter maritimus*	0	0	0	11% [1-20]	0	0	0	0	0
*Ralstonia mannitolilytica*	0	0	0	8%	0	0	0	0	0
*Serratia myotis*	0	0	0	11% [1-20]	13% [0-27]	0	0	0	0
*Stenotrophomonas rhizophila*	0	0	0	0	0	0	3%	0	0

pH and a_w_ were not immediately effected by slicing of the product (Table 1). Changes in pH at the end of the shelf-life period were slight (i.e. the unsliced vegetarian product logs had an average pH of 5.49, while in D28 samples an average pH of 5.36 was found) and mainly presented themselves in the vegetarian product logs sliced at 3 weeks.

### Culture-dependent identifications

Microbial identification and confidence intervals for the most abundant identifications were determined per type of sample (Table 2). In total, 205 colonies were identified. *Latilactobacillus sakei* was most abundant throughout the shelf-life period. It was found on the vegetarian product log as 10%, 100% and 100% of the isolates derived from PCA, RCA and MRS agar, respectively. At day-0, it was identified for 21%, 30% and 64% of the PCA, RCA and MRS agar isolates, respectively. Lastly, at the end of the shelf-life period, the species was identified for 47%, 59% and 67% of the PCA, RCA and MRS agar isolates. *Carnobacterium* spp., specifically *C. maltaromaticum*, were also detected both at day 0 and day 28, though rarely in the vegetarian product logs before slicing.

Gram-negative species were absent on the vegetarian product logs, yet they became noticeable in the product during slicing, as day-0 samples pointed towards the presence of 12 different species. However, at the end of the shelf-life period only two Gram-negative species were identified, i.e., *Pseudomonas versuta* and *Stenotrophomonas rhizophila*, both at merely 3% of the PCA isolates.

**Fig. 3:**
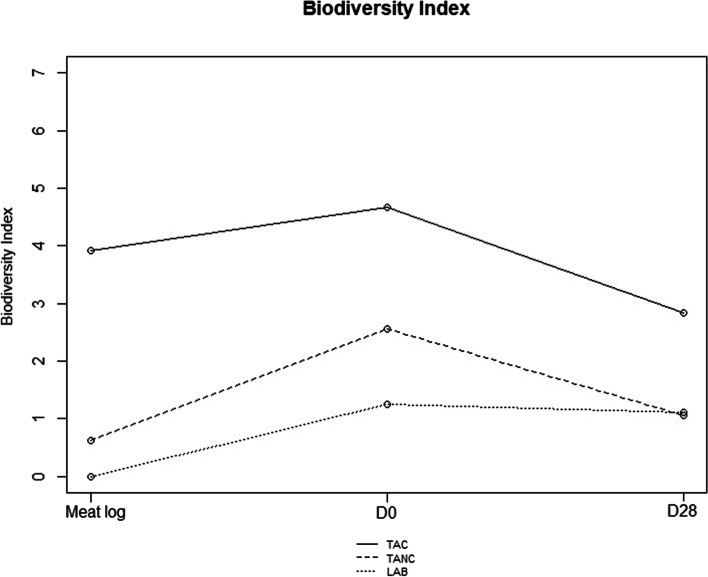
Biodiversity index. Biodiversity indexes for the number of identified species found for the total psychrotolerant and psychrotrophic aerobic counts (TAC), total psychrotolerant and psychrophilic anaerobic counts (TANC) and lactic acid bacteria counts (MRS agar) for curry-flavored vegetarian samples

Taken together, little consistency was found with respect to the identified species throughout the shelf-life period. As mentioned above, only *Latilactobacillus sakei* was consistently identified in these samples at high numbers. Figure 3 represents the changes in biodiversity indexes between isolates obtained from the vegetarian product log, the D0 samples and the D28 samples. An increase in biodiversity was noticeable between the vegetarian product log microbiota and D0 samples. Though, the species diversity decreased at the end of the shelf-life period, with specific species clearly prevailing.

Of the identified isolates from the slicer surfaces, 13% were identified as species that were also found in the actual product (Additional Fig. 1). The surface samples indicated an abundant presence of *B. thermosphacta* and *Staphylococcus equorum*, but these were not found in the product samples. *Carnobacterium divergens, C. maltaromaticum* and *Pseudomonas bubulae* were the only species found in both the environmental samples and the product samples.

### Metabarcoding

Metabarcoding analysis was performed on 30 samples. Nineteen samples (three of the unsliced vegetarian product log, eight at day 0 and eight at day 28) were retained for further analysis after rarefaction analysis, as a plateau phase was reached, meaning that sufficient sequence data was obtained for a reliable view on the bacterial ecosystem composition. The number of reads generated per remaining sample varied from 7,848 to 247,254 reads, with an average of 47,965 reads per sample (Additional Table 3). Yet, a significant amount of these reads were identified as chloroplasts or mitochondria. On average, 60% of the reads per sample were identified as chloroplast with an additional 10% being identified as mitochondria. Only two samples did not have high amounts of chloroplast and mitochondria: the unsliced vegetarian product log sample of VB5L1 (8 and 1%, respectively) and the day-28 sample of VB4L3 (1% and 0.2%, respectively).

**Fig. 4:**
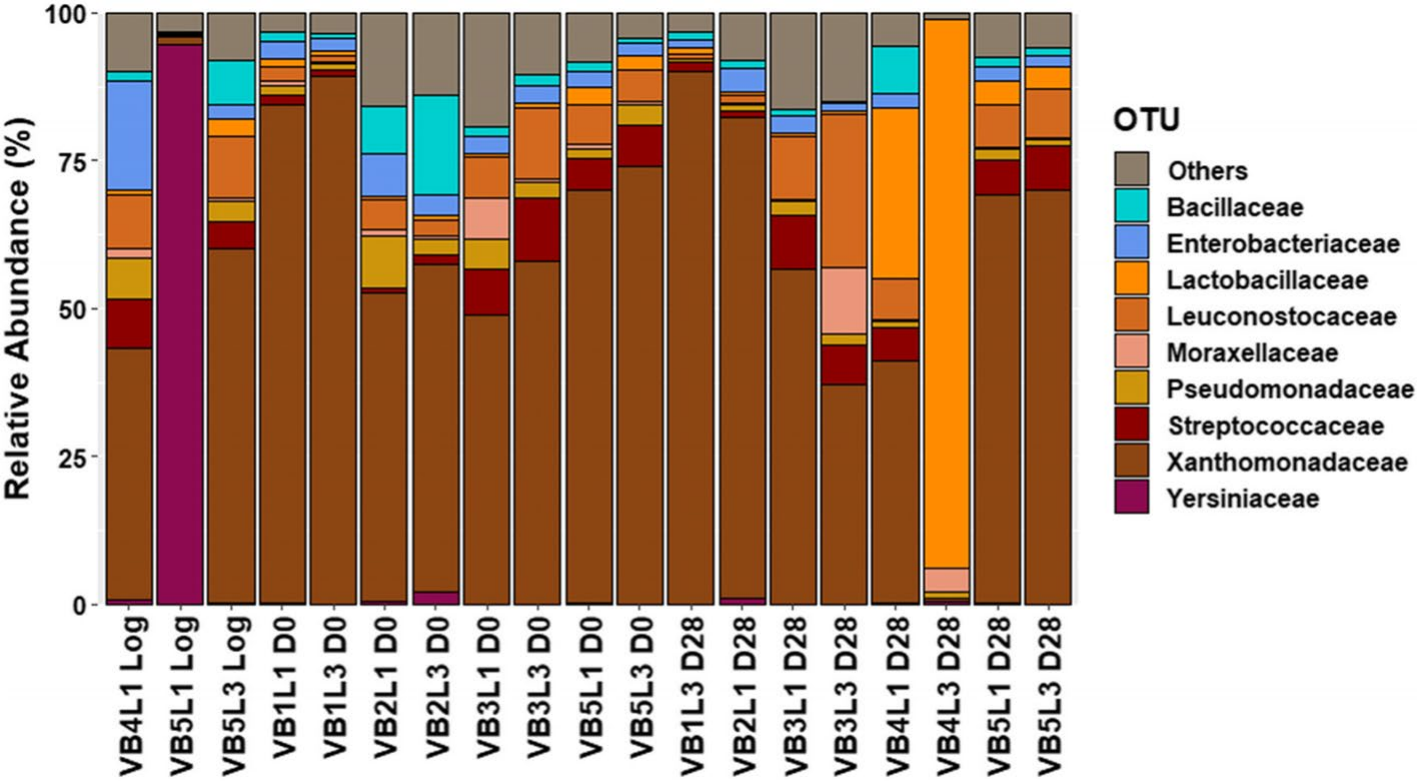
Cumulated histograms representing the relative abundance of taxa identified by metabarcoding at family levels. The category “Others” represents taxa present in <5% relative abundance. Following sample codes were used: V symbolizes vegetarian product. B indicates the batch number while L represents when an unsliced vegetarian product log was sliced (i.e. L1 and L3 represent vegetarian product logs sliced one and three weeks after production, respectively). D0 describes that the product was analyzed on the day of slicing, while D28 represent the product which was analyzed after 28 d of storage

**Fig. 5:**
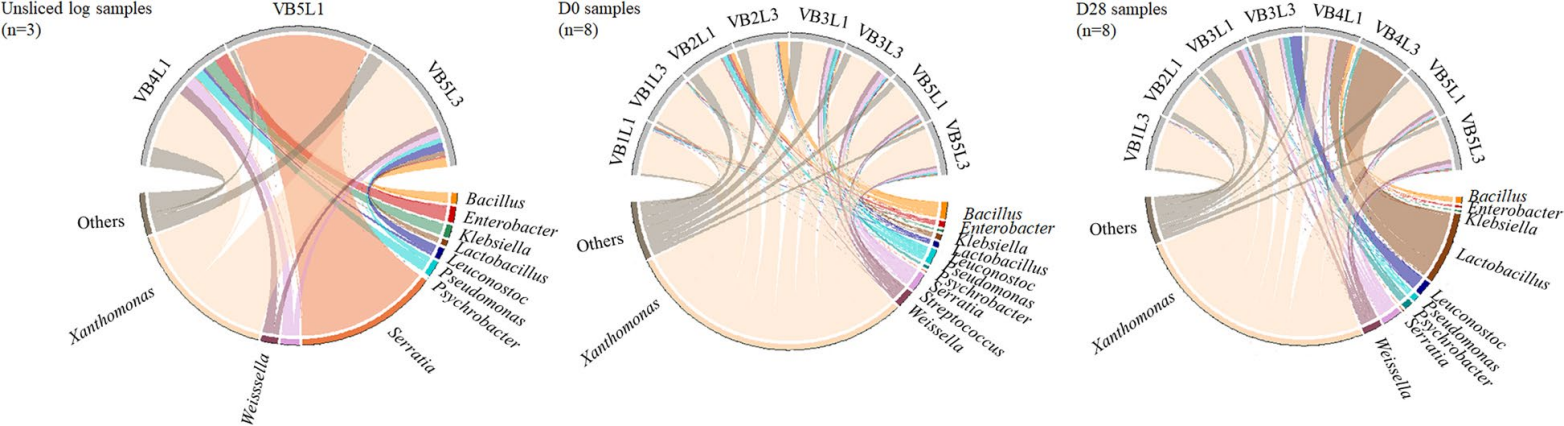
Chord diagrams representing the relative abundance of taxa identified by metabarcoding at genus levels. The category “Others” contains the taxa present in <5% relative abundance. Following sample codes were used: V symbolizes vegetarian product. B indicates the batch number while L represents when an unsliced vegetarian log was sliced (i.e. L1 and L3 represent vegetarian product logs sliced one and three weeks after production, respectively). D0 describes that the product was analyzed on the day of slicing, while D28 represent the product which was analyzed after 28 d of storage

Shannon-Wiener diversity and Chao1 richness indices were calculated (Additional Fig. 2). The relative population abundance at family level and at genus level, respectively, are shown in Figs. 4 and 5. The analysis presented a high relative abundance of *Xanthomonas* throughout the shelf-life period. In only two of the 19 retained samples, a different genus exceeded the relative abundance of *Xanthomonas*, namely *Serratia* in the unsliced vegetarian product log samples of VB5L1 and *Lactobacillus* in the day 28 sample of VB4L3. Also *Weissella* and *Streptococcus* were most often recovered within the LAB population. Both were recovered from one unsliced vegetarian product log sample, while *Streptococcus* was recovered from four D0 and five D28 samples; *Weissella* was recovered from three D0 and four D28 samples, in all cases at >5% relative abundance. Other, less often recovered genera in these samples were *Bacillus*, *Lactobacillus*, *Leuconostoc* and *Pseudomonas*.

## Discussion

Overall, the investigated meat product alternative was characterized by lower microbial loads at the end of its shelf-life period, at an average of 4.7 log CFU/g for TAC, than is typically the case for meat products under the same conditions (i.e. > 7 log CFU/g) [13, 25–27]. This could be due to differences in bacterial loads on the raw materials, lower pH values and/or due to the fact that the products were largely derived from chicken egg whites, containing antimicrobial compounds such as lysozyme [28]. Despite these quantitative differences in bacterial loads, the meat alternative presented the expected characteristic of microbial dominance by LAB under MAP conditions, which is also a common feature of MAP meat products [29–31]. Within the LAB population, the most important player was *Latilactobacillus sakei*, besides enterococci and carnobacteria.

The high prevalence of *Latilactobacillus sakei* has been reported before for vegetarian meat alternatives [3]. Selected strains of this species have the potential to be applied as bioprotective cultures, as they have demonstrated inhibition towards *B. thermosphacta, Leuconostoc mesenteroides* and *Listeria monocytogenes* in charcuterie products [32–34]. However, they can also induce significant acidification when they reach high levels, resulting in an unwanted acid taste and thus contributing to spoilage [33]. Also, they may produce ropey slime in cooked meat products under certain conditions, such as presence of sucrose, vacuum-packaging and a storage temperature of 8 °C [34].

In line with the fact that vegetarian meat alternatives may also contain enterococci such as *Enterococcus faecium* [3], enterococci were occasionally found. This occurred mostly at the end of the shelf-life period. Speculatively, the presence of these enterococci could originate from the use of curry, where their presence has been previously found at 2 log CFU/g [35]. The isolates identified in this study consisted of five different species, including *E. avium*, *E. devriesei*, *E. gilvus*, *E. malodoratus* and *E. viikkiensis*, but not *E. faecium*. The fact that these species were only found on PCA, RCA and MRS agar and not on S&B agar was probably due to the fact that most of them do not grow at 44 °C [36–38] and were therefore probably incapable of surviving the confirmation steps during the microbiological analysis as described in the Material and Methods section.

*Carnobacterium* spp. were consistently present throughout the shelf-life period, but were never able to reach a high abundance. Although *Carnobacterium* spp., in particular *C. divergens* and *C. maltaromaticum*, are known to be able to spoil meat in packaging with low traces of O_2_ [39–41], they are less likely to hold out at temperatures under 12 °C as they are less cold-adapted than meat associated lactobacilli [26]. The low yet measurable incidence of *C. inhibens* after slicing could be due to cross-contamination by a cooked chicken product [13], as both products are generally sliced on the same slicer, although this specific species has not been identified in environmental samples taken 8 months later.

With respect to bacteria other than LAB, *B. thermosphacta* was mostly not found despite being a common meat spoilage bacterium [42, 43]. It was only counted once on STAA (a sample at the end of the shelf-life period). This may be due to the low pH of the product, as the prevalence of this species is profoundly impacted by acidity [44–46], and/or to inhibition by *Lb. sakei* [32]. Gram-negative bacteria such as *Psychrobacter maritimus*, *Serratia myotis* and *Ralstonia mannitolilytica* were recovered just after slicing but were marginalized towards the end of the shelf-life period, as is often the case in chilled MAP goods [47–50]. This was likely due to the microaerobic to anaerobic atmosphere and the outgrowth of LAB that were able to outcompete Gram-negative SSOs [47, 48, 51].

To circumvent the problem of viable but non culturable microorganisms in microbiological mapping strategies [3], a complementary metabarcoding approach was used to complement the identification of cultivated isolates. As a downside, however, identification could only descend to genus level because only a small part of the 16 S rRNA gene was targeted [12]. To be able to predict the shelf-life period of a certain product, a more detailed insight into the presence of specific species and even strains in the present microbiota is often needed given their potential different abilities to spoil food [27, 51, 52]. A way to overcome this shortcoming would be to consider the full length 16 S rRNA gene by applying high-throughput, long read sequencing [53]. Another downside to the metabarcoding method is that it does not distinguish between viable and non-viable microbial cells [54]. Finally, comparing the results obtained by the culture-dependent and culture-independent methods revealed discrepancies, which indicates that results need to be carefully integrated.

 A first overall finding of the metabarcoding analysis was that there was not much variation observed in the recovered taxa and their individual relative abundances throughout the shelf-life period in contrast to the data from the culture-dependent analysis. Perhaps these limited observed variations were due to the mostly low bacterial loads on this type of product, even at the end of the shelf-life period. Exceptions in recovered microbiota included the unsliced vegetarian product log sample of VB5L1, with a high abundance of *Serratia*, the day-28 sample for VB3L3 (relatively high abundance of *Leuconostoc* and *Psychrobacter*) and both day-28 samples from batch 4: VB4L1 and VB4L3 (relatively high abundance of *Lactobacillus*). Of these 4 samples, the 2 samples that did not recover high relative abundances for *Xanthomonas* (product log VB5L1 and day 28 VB4L3) also had a low percentage of their ASVs identified as chloroplast and mitochondria. Another possible explanation for this variety could be differences in microbial loads: the product log for VB5L1 was below enumeration limit for TAC, TANC and LAB on both MRS and M17 medium, while both day-28 samples for batch 4 had the highest enumerations found for these parameters (e.g. 7.1 and 8.1 log CFU/g TAC).

Another contrast was the discovery of a potential strong presence of *Xanthomonas* through-out the shelf-life period with the metabarcoding approach, whereas this genus was not found using the culture-dependent method. This can potentially be explained, though, by the presence of xanthan gum (or E415) in the product, which is used as a stabilizing, thickening or emulsifying agent, industrially produced by *Xanthomonas campestris* strains [55]. Indeed, when the obtained *Xanthomonas* reads were aligned against the EzBioCloud database, a hit for *X. campestris* was obtained with 99.77-100% identity. Although *Xanthomonas* can be a factor in food spoilage, this is usually the case for fruits and vegetables [55]. If recovery of *Xanthomonas* DNA was due to the presence of xanthan gum, no viable *Xanthomonas* cells were most likely present, which corroborates the inability to identify *Xanthomonas* using the culture-dependent method.

A third contrasting finding was that the metabarcoding data indicated *Weissella* and *Streptococcus* as the main LAB, which were not found as species using the culture-dependent method. The latter instead emphasized the presence of *Latilactobacillus*, *Leuconostoc* and *Carnobacterium* species, commonly found in chilled MAP charcuterie [39, 56, 57]. Yet, *Lactobacillus* (which could also include the genus *Latilactobacillus*) was however recovered at a high relative abundance in two day-28 samples taken from the same batch (at 29 and 93% in VB4L1 and VB4L3, respectively), which could explain the high amount of isolates identified as *Latilactobacillus sakei* at the end of the shelf-life period. Coincidentally, both day-28 samples also had the highest total aerobic counts at 7.1 and 8.1 log CFU/g, while the average TAC for this product type was 4.7 log CFU/g. This lower TAC at the end of the shelf-life period was in accordance with earlier findings in vegetarian products that were also partly made up out of egg white powder (Geeraerts et al., 2020). *Weissella* is known to occur on MAP meats and poultry and can cause slime and/or greening due to the formation of H_2_O_2_ [39, 56, 58]. The genus has also been recovered in cooked ham after thermal processing [26]. The presence of *Streptococcus* was more puzzling, as streptococci are not generally food-associated organisms or food spoilers, with the benign exception of *S. thermophilus* in certain fermented dairy products (e.g. yoghurt or certain Italian cheeses) [59]. This species is, however, more thermophilic and heat-resistant [60] and thus there is the possibility that it survives the applied heat treatments.

In conclusion, it can be stated that generally, the main SSOs in the type of product investigated were identified as *Lb. sakei* and, to a lesser extent, *C. divergens* and *C. maltaromaticum*, though variation can be seen between batches (in the case of high relative abundances of *Lactobacillus* recovered by metabarcoding in both samples retrieved from batch 4 at the end of the shelf-life period) or individual samples (e.g. the high relative abundance of *Serratia* recovered in the vegetarian product log in 1 sample of batch 5, which was not recovered in the other sample). Also, reliable interpretation of the applied metabarcoding method proved difficult in this type of product, as there was too much interference against a low bacterial load from both chloroplast DNA and the presence of *Xanthomonas* DNA due to the use of xanthan gum as a stabilizing agent. A way to circumvent these issues might be the application of a bacteriological enrichment step before metabarcoding. This method also has its downsides, as it will select only a subset of culturable bacteria and hence will change the initial structure of the microbiota composition. The advantage of adding this step is that a rare species of interest might be detected where it was before missed [61]. Combining 16 S metabarcoding with and without enrichment could be applied to accurately analyze this type of matrix. As the low bacterial loads present on these products are thought to be a limiting factor in applying the metabarcoding technique, a preliminary bacteriological enrichment step might be used but this will change the initial structure of the microbiota composition. Alternatively, selective depletion of matrix DNA through osmotic lysis and treatment with propidium monoazide could be explored. This approach has already been successful on clinical samples where dominating host DNA obscures changes in microbial populations as few DNA sequence reads had microbial origins [62].

## Supplementary Information

**Additional file 1.**

## Data Availability

The sequencing data have been deposited in the NCBI SRA database to the BioProject accession number PRJNA734172.
