# Supplementary material for: To culture or not to culture: careful assessment of metabarcoding data is necessary when evaluating the microbiota of a modified-atmosphere-packaged vegetarian meat alternative throughout its shelf-life period
Source: BMC Microbiol. 2022 Jan 25;22:34. doi: 10.1186/s12866-022-02446-9 (PMC8788083; doi:10.1186/s12866-022-02446-9)
Supplement: Supplementary file 1 — Additional file 1. [file 12866_2022_2446_MOESM1_ESM.docx]

**Additional files**

Additional Table 1: Log(CFU/g) values at different production and sampling stages [unsliced vegetarian product log, after slicing (D0) and at end of the shelf-life period (D28)] for total psychrotolerant and psychrophilic aerobic (TAC) and anaerobic counts (TANC) and for lactic acid bacteria counts (LAB). For microbial enumerations, means are given are given as log(CFU/g) values when there was at least one sample above enumeration limit and standard deviations are given as log(CFU/g) values when there was more than one sample above enumeration limit. In other instances, the value was assigned as ‘not applicable’ (na).

|  |  | **TAC** | | | **TANC** | | | **LAB (MRS)** | | | **LAB (M17)** | | |
| --- | --- | --- | --- | --- | --- | --- | --- | --- | --- | --- | --- | --- | --- |
|  |  | **Unsliced log** | **D0** | **D28** | **Unsliced log** | **D0** | **D28** | **Unsliced log** | **D0** | **D28** | **Unsliced log** | **D0** | **D28** |
| **1 week** | Batch 1 | <1 | 3,0 | 3,5 | <1 | 3,2 | <1 | 1,3 | 3,2 | 2,1 | <1 | 2,5 | <1 |
|  | Batch 2 | 4,2 | 3,8 | 3,8 | 3,1 | 2,6 | 3,5 | <1 | <1 | 3,2 | <1 | 1,3 | 3,9 |
|  | Batch 3 | <1 | 4,3 | 4,5 | <1 | 3,8 | 4,7 | <1 | 3,7 | 4,6 | <1 | 4,1 | 4,6 |
|  | Batch 4 | <1 | 2,9 | 7,1 | 3,6 | 3,5 | 6,6 | <1 | <1 | 6,6 | <1 | 2,0 | 6,9 |
|  | Batch 5 | <1 | <1 | <1 | <1 | <1 | <1 | <1 | <1 | <1 | <1 | <1 | <1 |
|  | *Mean* | *4,2* | *3,5* | *4,7* | *3,4* | *3,3* | *4,9* | *1,3* | *3,4* | *4,1* | *na* | *2,5* | *5,1* |
|  | *Standard deviation* | *na* | *0,7* | *1,6* | *0,3* | *0,5* | *1,6* | *na* | *0,3* | *1,9* | *na* | *1,2* | *1,6* |
| **3 weeks** | Batch 1 | <1 | 3,3 | 2,8 | <1 | 2,6 | 2,2 | <1 | 2,5 | 3,1 | <1 | 3,2 | 3,2 |
|  | Batch 2 | 1,3 | 2,0 | 6,8 | <1 | 2,6 | 6,0 | <1 | <1 | 6,5 | 2,7 | 1,8 | 6,6 |
|  | Batch 3 | 1,8 | 1,0 | 4,5 | <1 | <1 | 4,4 | 3,3 | <1 | 4,6 | 1,7 | 1,3 | 4,6 |
|  | Batch 4 | 3,3 | 4,3 | 8,1 | 2,9 | 4,3 | 8,7 | 2,6 | 4,4 | 7,8 | 2,6 | 4,3 | 7,7 |
|  | Batch 5 | 1,3 | 1,3 | 1,6 | <1 | <1 | <1 | <1 | <1 | <1 | <1 | 1,0 | 1,6 |
|  | *Mean* | *1,9* | *2,4* | *4,8* | *2,9* | *3,2* | *5,3* | *3,0* | *3,5* | *5,5* | *2,3* | *2,3* | *4,7* |
|  | *Standard deviation* | *0,9* | *1,4* | *2,7* | *na* | *1,0* | *2,7* | *0,5* | *1,3* | *2,1* | *0,6* | *1,4* | *2,5* |

Additional Table 2: Log(CFU/g) values at three different production stages [unsliced vegetarian product log, after slicing (D0) and at the end of the shelf-life period (D28)] for presumptive yeast and fungi (OGYE), presumptive sulphite-reducing clostridia (TSC), presumptive Enterobacterales (VRBGA), presumptive *Bacillus cereus* (MYP), *Brochothrix thermosphacta* (STAA) and presumptive *Enterococcus* (Slanetz & Bartley). The log(CFU/g) value was given when only one sample was above enumeration limit, or the value was assigned as ‘not applicable’ (na) when all samples were below enumeration limit.

| **OGYE** | | **TSC** | | | | **VRBGA** | | **MYP** | | | | **STAA** | | | **Slanetz & Bartley** | | | |  |  |
| --- | --- | --- | --- | --- | --- | --- | --- | --- | --- | --- | --- | --- | --- | --- | --- | --- | --- | --- | --- | --- |
|  | | N^a^ | Log (CFU/g) | N | | | Log (CFU/g) | N | Log (CFU/g) | N | | | Log (CFU/g) | N | Log (CFU/g) | | N | | Log (CFU/g) | |
| *1 week* |  | |  | |  |  | |  |  | |  |  | |  | |  | |  | |  |
| Unsliced log | | 1 | 1.0 | 0 | | | na | 0 | na | 0 | | | na | 0 | na | | 0 | | na | |
| D0 | | 1 | 1.6 | 0 | | | na | 1 | 1.0 | 0 | | | na | 0 | na | | 0 | | na | |
| D28 | | 0 | na | 0 | | | na | 0 | na | 0 | | | na | 0 | na | | 0 | | na | |
| *3 weeks* |  | |  | |  |  | |  |  | |  |  | |  | |  | |  | |  |
| Unsliced log | | 0 | na | 0 | | | na | 0 | na | 0 | | | na | 0 | na | | 0 | | na | |
| D0 | | 0 | na | 0 | | | na | 0 | na | 0 | | | na | 0 | na | | 0 | | na | |
| D28 | | 1 | 1.8 | 0 | | | na | 0 | na | 0 | | | na | 1 | 2.0 | | 0 | | na | |

^a^N, number of samples where enumerations were above enumeration limit

Additional Table 3: The number of generated sequence reads, given for each sample which was retained after rarefaction curve analysis.

| **Sample** | **Number of reads** | **Sample** | **Number of reads** |
| --- | --- | --- | --- |
| VB1L1 D0 | 44798 | VB4L1 Log | 13756 |
| VB1L3 D0 | 7848 | VB4L1 D28 | 46825 |
| VB1L3 D28 | 22230 | VB4L3 D28 | 247254 |
| VB2L1 D0 | 104039 | VB5L1 log | 51708 |
| VB2L1 D28 | 25117 | VB5l1 D0 | 30415 |
| VB2L3 D0 | 22070 | VB5L1 D28 | 20543 |
| VB3L1 D0 | 104368 | VB5L3 log | 16504 |
| VB3L1 D28 | 9434 | VB5L3 D0 | 100573 |
| VB3L3 D0 | 10658 | VB5L3 D28 | 11956 |
| VB3L3 D28 | 9434 |  |  |


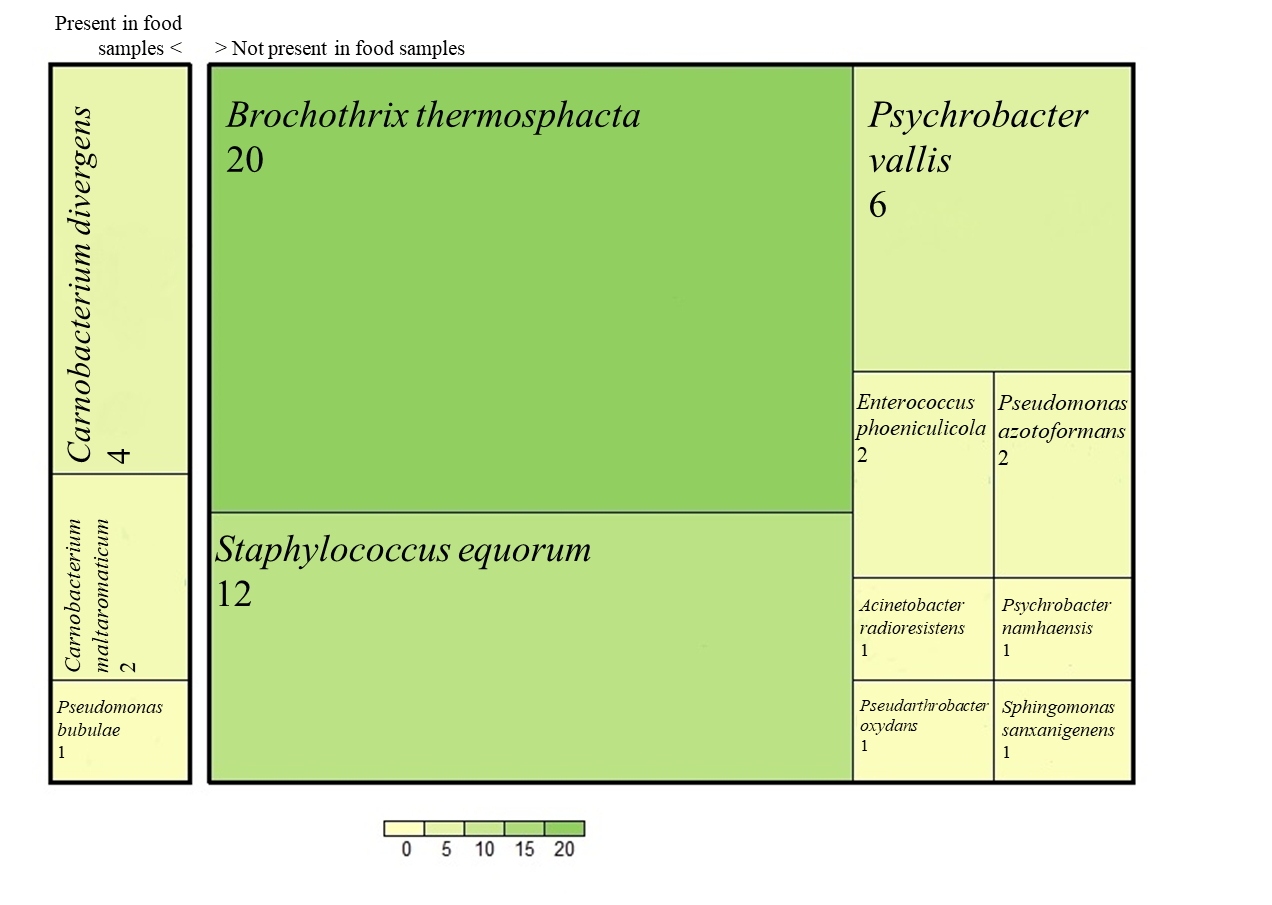


Additional Figure 1: Genus and species identity of isolates. Genus and species identity of isolates (n=53) from PCA, RCA and MRS agar media for food contact surface swab samples obtained from the vegetarian meat alternative slicer.


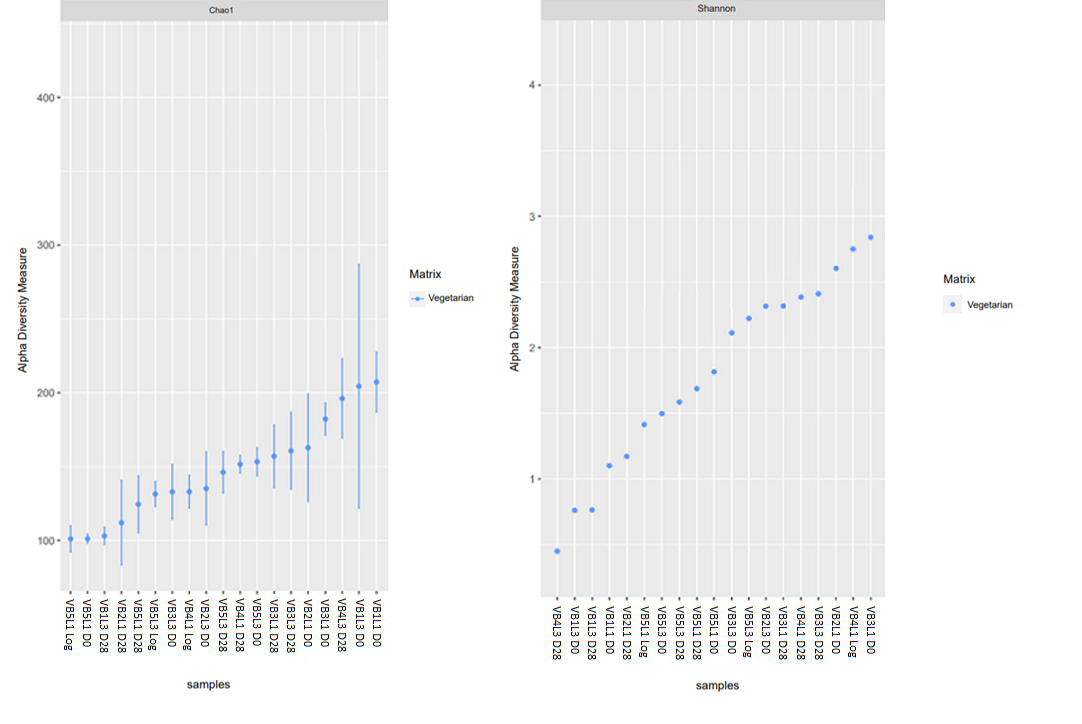


Additional Figure 2: Chao1 and Shannon-Wiener diversity richness indices. Sample codes: V stands for vegetarian product. B is the batch number while L indicates when an unsliced vegetarian product log was sliced (*i.e.* L1 and L3 represent vegetarian product logs sliced one and three weeks after production, respectively). D0 indicates that the product was analyzed on the day of slicing, while D28 indicates that the product was analyzed after 28 d of storage.
